# What About the Environment? How the Physical Activity–Related Health Competence Model Can Benefit From Health Literacy Research

**DOI:** 10.3389/fpubh.2021.635443

**Published:** 2021-04-16

**Authors:** Johannes Carl, Eva Grüne, Klaus Pfeifer

**Affiliations:** Department of Sport Science and Sport, Friedrich-Alexander University Erlangen-Nürnberg, Erlangen, Germany

**Keywords:** physical activity, structure, interaction, PAHCO model, physical literacy

## Abstract

Research cultivates a multitude of frameworks, models, and theories with different determinants internal and/or external to the individual contributing to the understanding and explaining of physical activity levels. The physical activity–related health competence (PAHCO) model can be located at the interface between research of health literacy and physical activity. Because of its primary person orientation, however, the model has not yet undergone discussions on the relevance of the environment. Encouraged by the developments in the area of health literacy, the goal of the present perspective article was to stimulate some initial reflections on potential solutions for the competence–environment relationship within the PAHCO model. We extracted three potential solutions for this issue. Dubbed the solution of integration, we first discussed that the PAHCO model could be placed into overarching, more holistic, and abstract models of health-enhancing physical activity, such as the capability approach or the socioecological model. Applying a solution of elaboration, researchers could second substantiate existing components of the PAHCO model, such as control competence or self-regulation competence, by further explanations. Characterizing the solution of extension, it would third be possible to introduce (a) separate competence component(s) that highlight(s) the manageability of the environment, for instance, by establishing a (socio)ecological competence. The article concludes with a short overview of potential empirical approaches, given their potential to assist researchers in identifying preferences for the theoretical advancement and to put the development on a stronger evidence base.

## Introduction

### Health Promotion and the Role of Physical Activity

Because health is regarded as the precious asset in today's society, being healthy or behaving accordingly is of great importance for every individual. However, maintaining and promoting health are not only an individual concern but also a public health issue and is therefore on the agenda of research, policy, and practice. Supported by the considerable accumulation of evidence (1, 2), physical activity has been identified as an important resource for the maintenance or improvement of health. Hence, initiatives addressing physically (in)active lifestyles have gained increasing importance over the last decades [e.g., Global Action on Physical Activity 2018–2030 (GAPPA), see (3)]. Recognizing the importance of physical activity and launching initiatives for its promotion are accompanied by the question of which interventions are most effective. However, this question is difficult to answer in the light of the available evidence. Nevertheless, when developing interventions to promote physical activity in individuals, it is necessary to understand why some people are physically active and others not (4).

### Person-Related Approaches for Physical Activity: The Physical Activity–Related Health Competence Model as an Example

As highlighted by a current historical synthesis, research cultivates different theoretical approaches to explain changes in human physical activity behavior (5). In this context, theoretical concepts addressing individual competences or literacy have recently become the focus of discussion with a high relevance also for behavior change. The notion of competence has its scholarly roots in the psycholinguistics but has received most attention in the educational sciences (6). The term implicates that individuals should possess or acquire latent dispositions, delimitable from actual performance (7, 8), which empower them to lead a certain lifestyle (9). In temporal regards, competence detaches from the short-term horizon and rather stresses that qualifications and resources can be maintained over a longer period (10). Taken together, these conceptual conditions make the notion of competence attractive for the long-term development of health-enhancing physical activity (HEPA), for behavior change interventions, and for physical activity promotion and health promotion in particular.

**Figure 1:**
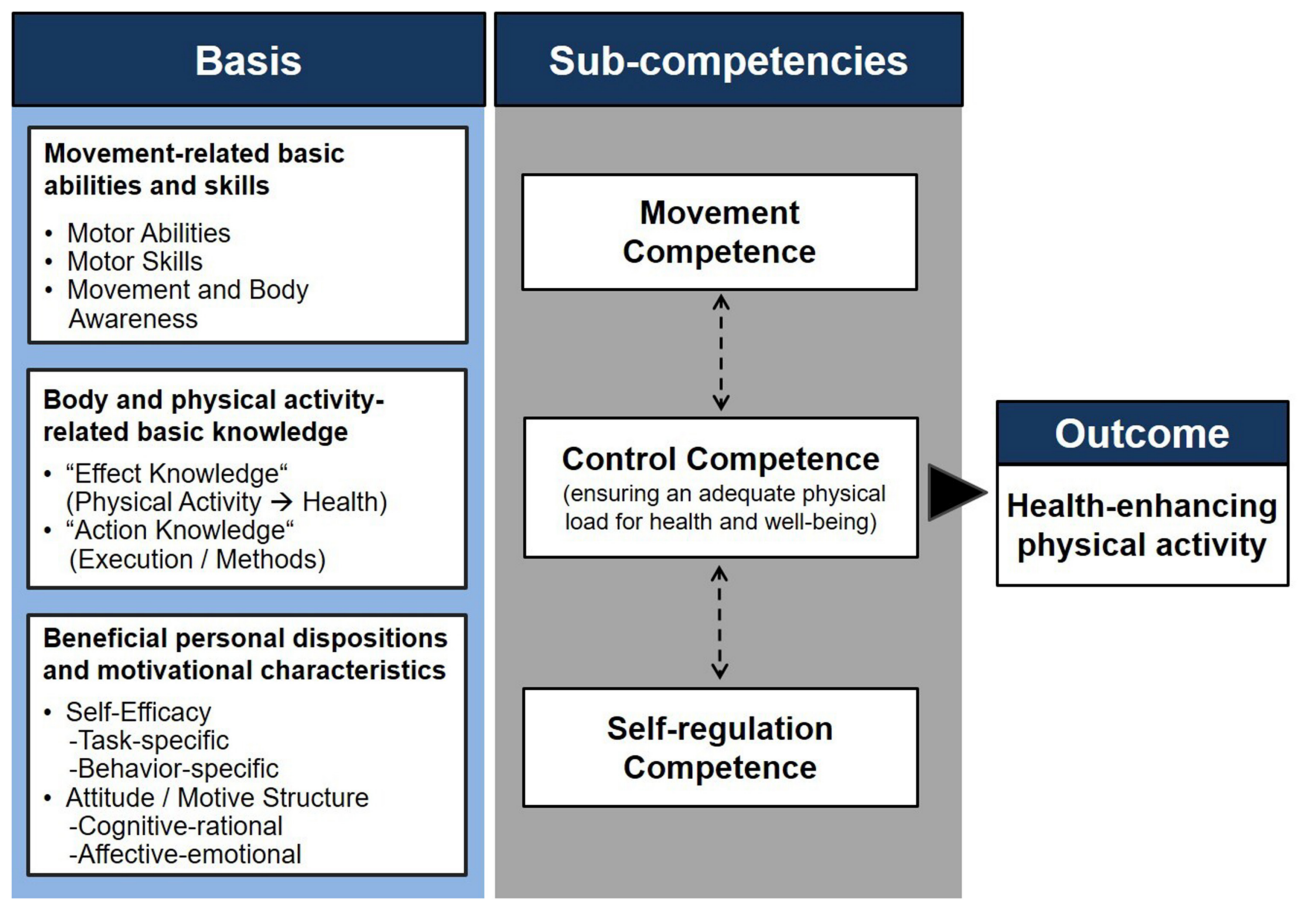
The physical activity–related health competence (PAHCO) model (11, 12).

As one of these approaches drawing on the general ideas of competence, the physical activity–related health competence (PAHCO) model (9, 11) posits that individuals require three integrated subcompetences to lead a healthy, physically active lifestyle (Figure 1). First, people need movement competence, which describes the direct motor-related requirements allowing individuals to master activities of daily living and to participate in planned exercise. As a motivational–volitional requirement, the second area, self-regulation competence, guarantees the regular execution of physical activities necessary to induce adaptations for health. As more of a qualitative dimension, the third area, control competence, does not merely follow the formula “the more, the better.” Rather, this area ensures that the loads and characteristics of physical activity and exercise meet the individual's requirements to promote both physical (e.g., adequate exercise stimulus, avoidance of overload) and psychological (e.g., avoidance of sports addiction, promotion of mental well-being) health. These three subcompetences are, in turn, the result of the integration of knowledge, abilities/skills, and attitudes (13)—the so-called basic elements [for an extensive outline, see (9, 11)].

The PAHCO model with its multidimensional and integrative view has recently been used in different target groups and settings attributable to both prevention (12, 14–19) and rehabilitation (12, 20–23). However, when reviewing the first conceptual–theoretical articles, it becomes obvious that environmental factors hardly play any role within this competence approach (11, 24). Congruent with the function of models in general (25) and in line with a mostly person-focused view of competence (6), PAHCO adopts a selective perspective on a phenomenon through the concentration on individual determinants for a healthy, physically active lifestyle. This may partially explain why previous empirical articles on PAHCO revealed promising yet not fully satisfactory levels of explanation for indicators of HEPA. Depending on the target group, PAHCO could explain between 10 and 53% of the variance in indicators of PA and health (11, 15, 26). In any case, the PAHCO model does not yet represent those influencing factors outside the person or the interaction of the individual with the environment to achieve beneficial levels of physical activity. Therefore, there is potential for the PAHCO model to better harmonize with central assumptions of the socioecological approach for health (27). The socioecological approach has experienced a considerable growth trajectory within behavior change literature on physical activity over the last two decades (5), which can be explained, to a large extent, by the fact that the corresponding models consider different explanatory levels simultaneously, from the individual to the environment (28). In one of these endeavors, for instance, Bauman and colleagues (29) listed several determinants at the individual, behavioral, social, environmental, and political level that contribute to explaining physical activity. In this regard, the latest discussions of PAHCO focused on the individual and behavioral levels within this differentiation, whereas the social, environmental, and political levels have not been addressed in detail so far.

### The Role of the Environment in Health Literacy Research

With its consideration of person-related determinants for health, the PAHCO model shows significant parallels and overlaps to the research field of health literacy (9). According to a widespread definition, health literacy comprises people's qualifications “to access, understand, appraise, and apply health information in order to make judgments and take decisions in everyday life” (30). The information aspect, which has been extracted separately in a content analysis across different studies (30), stands at the core of the concept and exerts an instrumental (“in order to”) value by determining subsequent evaluations (“judgments”) and decisions. Despite the emphasis of the information aspect and the associated importance of cognitive processing (including perceptions, understanding, appraising, and the deduction of plans and intentions for action), a multitude of research endeavors underlined the social embeddedness of the individual's health literacy (31–34). The widespread integrated model of health literacy comprises social as well as environmental determinants, and, following a public health perspective, it welcomes population-level efforts, thereby postulating participation and equity as potential outcomes (30). Accordingly, the scientific discussion on the relevance of the environment has gained momentum (35–37). For instance, the research activities have yielded the construct of organizational health literacy as a beneficial characteristic of institutions or systems supporting people to navigate, understand, and use information and services to take care of their health (34, 38, 39). The considerations of the environment also permeated the action plans of several countries (40), which provide national efforts with an adequate framework for health promotion. Taken together, the emphasis of social embeddedness and the release of action plans reflect that health literacy is no longer the sole responsibility of individuals but is also an issue of the general public and thus a matter of political acting. These tendencies have turned health literacy into a concept that has detached from the mere person-relatedness (41).

In this regard, health literacy research, as a related research field being one step ahead, might serve as an example for showing how successive discussions on the role of the environment may stimulate the advancement of a person-related concept. Inspired by the developments of the adjacent health literacy field, the present perspective article provides some initial considerations regarding potential solutions how to better account for the relevance of the environment within the PAHCO model. In the present article, PAHCO is used as a specific example for person-related approaches for physical activity. In the long run, this journey toward a more holistic approach may culminate in a better convergence of person-related and environmental determinants for HEPA, as requested by GAPPA (3) and biopsychosocial integration efforts (42). From an interventional perspective, this may lead to a better knowledge of social determinants and implementation conditions of HEPA or, depending on the solution preferred, to an activity-related empowerment of individuals interacting with the environment. Ultimately, we derived three potential solutions for the PAHCO model; an overview is given in Table 1.

**Table 1:** An overview of the three potential solutions.

**Solution**	**Abstract characterization**	**Theoretical consequence for the PAHCO model**	**Associated empirical consequence for the PAHCO model**
(1) Integration	The model is integrated into a broader framework that considers both individual and environmental factors	The PAHCO model has to be integrated into a broader framework (e.g., the socioecological model or the capability approach)	The existing operationalizations of PAHCO must fit within the (operationalizations of the) broader framework
(2) Elaboration	Existing model components are basically compatible with environmental factors; however, they must still be elaborated conceptually	The subcompetences (movement, control, and self-regulation competence) of the PAHCO model have to be elaborated by discussing the role of the environment	Authors should develop an operationalization of the new component facet, which should then empirically fit to the theoretically postulated (existing) model component
(3) Extension	The conceptualization of model components is not compatible with the environment; a numerical extension of model components is undertaken	Introduction of a fourth PAHCO subcompetence (e.g., potentially dubbed “socioecological competence”)	Authors should develop an operationalization of the new model component, which should (i) delimit from the other components and (ii) provide a substantial explanation for relevant outcomes

*PAHCO, physical activity–related health competence*.

## Potential Solutions for the Pahco Model

### Integration

As a first solution, it could be possible to embed PAHCO into a broader, ideally well-established, framework underscoring the interaction between the individual and the environment. For example, researchers could define PAHCO as constituting the intrapersonal level within the social ecological model of physical activity (28). The intrapersonal factors, in turn, interact with the surrounding layers of the model (27). As a second example, it might be possible to integrate the PAHCO model into the health capability approach (43–45), which relies on Giddens' (46) dualistic assumptions of structure and agency. When choosing this solution of integration, researchers may detail the theoretical integration [which has already been partially caught up in the context of PAHCO, see (9)] in order to ensure that both approaches can be brought together. In this context, theory of science calls for ensuring commensurability between model components (47, 48). This solution, however, bears the risk of increased model complexity and even theoretical oversaturation, as supported by a meta-analysis demonstrating that physical activity interventions are less efficient if they are based on a combination of theories instead of a single theory (49).

### Elaboration

As a second solution, researchers could incorporate the manageability of environmental influences into existing competence components. This solution presupposes that existing conceptualizations of PAHCO components are basically compatible with the intended incorporation. Notably, in this case, it is not the environment *per se* that enters the competence structure model of PAHCO. In line with an interactionist understanding of competences (50, 51), it is rather the individual manageability of social, structural, environmental, or political demands and challenges that this model solution considers essential for the execution of HEPA. In any way, this solution calls for an elaboration of conceptual descriptions of existing competence components. More specifically, these descriptions should target facets of existing components that reflect the manageability of environmental demands, for instance, if the physical activities must be executed in a regular manner (self-regulation competence) or if adequate physical loads must be chosen for physical health and psychological well-being (control competence). Currently, some single model-related descriptions appear promising, as they address the overcoming of barriers and mention the importance of situation-adequate reactions (11, 24), and may thus serve as a starting point for further elaboration.

### Extension

If the management of structural–environmental demands is not sufficiently compatible with or captured by existing model components, a final option may consist in formulating an additional competence component into the PAHCO model. Within the three competence–environment relationships, this option can be referred to as to the solution of extension. For instance, a fourth competence component could be introduced at the subcompetence level of PAHCO, potentially denoted as (socio)ecological competence. This new competence component could be primarily formed by the coupling of social and environmental perceptions with other beneficial dispositions, such as self-efficacy (27, 52). However, this solution makes it necessary to find arguments that (a) justify the use of the new construct in the context of HEPA, e.g., (socio)ecological competence, (b) empirically support an effect of this component on indicators of HEPA, (c) underline the conceptual gain beyond the established model components (ideally supported by data showing discriminant/incremental validity), and (d) bring the new concept to the same theoretical level as the remaining model components, including the integrative and interrelated ideas.

## Discussion and Future Directions

This perspective article worked out three potential solutions, using the PAHCO model as an example, how the role of the environment might be considered in competence-oriented endeavors for physical activity. The solution of integration section Integration relies on the theoretical characteristics of an overarching framework or theory, whereas the solutions of elaboration section Elaboration and extension section Extension incorporate the manageability of environmental demands into potentially commensurable components through the specific lens of competence. The three solutions might have both theoretical and practical values for the field of physical activity promotion and health promotion and hence can be subject of future discussions. Of course, the present contribution does not claim to present an exhaustive list of solutions. For instance, it might be conceivable to include environmental factors pragmatically to multivariate analyses with person-related measurements. This solution bridging the two pillars of individual and environment, however, remains theoretically expandable, as the plea for conceptual integration and compatibility/commensurability remains unaddressed.

Ideally, the “new” or evolved theoretical model finds its support in empirical data as well. Opposed to a confirmatory approach, empirical data can already be used at an earlier stage of theory advancement. Identifiable as an explorative approach, researchers could develop valid and reliable operationalizations of “manageability of the environment.” Subsequently, it could be tested whether the new measurements (a) can be rather assigned to already existing model facets (e.g., self-regulation competence) or (b) whether they form a separately extractable subcompetence factor. In this specific case, statistical model comparisons using a validated, hierarchical assessment instrument (12, 26) could give researchers an initial hint of whether to prefer the (a) elaborating or (b) extending solution of PAHCO.

The solution preferred, in turn, determines the implications that are drawn when the ideas of the advanced model are translated into an intervention. The solution of integration may more strongly shift the focus from the individual to the environment. Through the lens of this solution, modifications targeting the organizational or social level appear promising when they significantly improve the conditions for the promotion of competences. The solutions of elaboration and extension, in contrast, would put more emphasis on the individual management and perceptions of environmental demands. Therefore, the associated measures could substantially complement person-centered approaches for physical activity, such as behavioral counseling (53).

In summary, the advancement of person-related concepts, which have found broad acceptance in behavior change literature, presents a difficult and complex matter. Nevertheless, theoretical advances underpinned by empirical arguments might have the potential to approach the requested amalgamation of person-related and environmental factors for physical activity, unified under the integrative perspective of competences. We assume that discussions on the role of the environment are urgent, leading to an extension of existing perspectives, such as adopted by the PAHCO model. In this regard, health literacy research can be ascribed a pioneering role as the field was successful in systematically advancing such discussions.

## Conclusion

The present article aimed at transcending the person-related concept of PAHCO by stimulating reflections on the role of the environment for HEPA. With the integrating, elaborating, and extending solutions, the authors suggested three options how to potentially guide the advancement of such a concept. Future research articles, either dealing with a person-related HEPA concept or with PAHCO in specific, are invited to use the present perspective as a starting point for ongoing, more detailed conceptualizations. Ideally, researchers find both theoretical and empirical arguments to justify their extension strategy.

## Data Availability Statement

The original contributions presented in the study are included in the article/supplementary material, further inquiries can be directed to the corresponding author/s.

## Author Contributions

JC initiated/promoted the advancement of theory, drafted/revised the manuscript, and organized the discussion process. EG wrote a part of the introduction and contributed substantially to the theoretical discussions. KP provided important feedback and supervised the whole advancement of theory. All authors contributed to the article and approved the submitted version.

## Conflict of Interest

The authors declare that the research was conducted in the absence of any commercial or financial relationships that could be construed as a potential conflict of interest.
